# Flexible Measurement of Bioluminescent Reporters Using an Automated Longitudinal Luciferase Imaging Gas- and Temperature-optimized Recorder (ALLIGATOR)

**DOI:** 10.3791/56623

**Published:** 2017-12-13

**Authors:** Priya Crosby, Nathaniel P. Hoyle, John S. O'Neill

**Affiliations:** ^1^MRC Laboratory of Molecular Biology

**Keywords:** Molecular Biology, Issue 130, Bioluminescent reporters, circadian, ALLIGATOR, PERIOD2, luciferase, perfusion

## Abstract

Luciferase-based reporters of cellular gene expression are in widespread use for both longitudinal and end-point assays of biological activity. In circadian rhythms research, for example, clock gene fusions with firefly luciferase give rise to robust rhythms in cellular bioluminescence that persist over many days. Technical limitations associated with photomultiplier tubes (PMT) or conventional microscopy-based methods for bioluminescence quantification have typically demanded that cells and tissues be maintained under quite non-physiological conditions during recording, with a trade-off between sensitivity and throughput. Here, we report a refinement of prior methods that allows long-term bioluminescence imaging with high sensitivity and throughput which supports a broad range of culture conditions, including variable gas and humidity control, and that accepts many different tissue culture plates and dishes. This automated longitudinal luciferase imaging gas- and temperature-optimized recorder (ALLIGATOR) also allows the observation of spatial variations in luciferase expression across a cell monolayer or tissue, which cannot readily be observed by traditional methods. We highlight how the ALLIGATOR provides vastly increased flexibility for the detection of luciferase activity when compared with existing methods.

**Figure:**
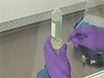


## Introduction

The use of luciferases as reporters of gene expression and protein activity has become a popular technique in molecular and cellular biology research. This is true in the circadian field, where the kinetics of firefly luciferase synthesis and catalytic inactivation are particularly well suited to reporting the longitudinal changes in gene expression that occur over the approximately 24 h circadian cycle. As such, luciferase is employed as a circadian reporter across a wide range of organisms, including fungi, plants, flies, and mammals^1^,^2^,^3^,^4^.

When quantifying circadian gene expression *in vitro*, a photomultiplier tube (PMT) is commonly used to record the bioluminescent signal. PMT-based measurements have limited flexibility however, usually being restricted to a pre-determined plate or dish size. It is also not possible to collect any spatial information from samples monitored using a PMT, which can lead to a loss of information when imaging samples that show spatial variation in luciferase expression. Furthermore, as the PMT and associated electronics are prone to malfunction when exposed to the humidified environment of a standard cell culture incubator, longitudinal luciferase recording using PMTs is always performed in non-humidified incubators. In consequence, cell culture dishes must be sealed air-tight to prevent moisture loss through evaporation and the culture media must therefore be buffered with 3-(*N*-morpholino)propanesulfonic acid (MOPS) or 4-(2-hydroxyethyl)-1-piperazineethanesulfonic acid (HEPES), rather than the CO_2_/bicarbonate buffering system that functions *in vivo *and is used routinely in mammalian tissue culture.

As a result of these limitations, measurement of bioluminescence by PMTs usually places tight restrictions upon the conditions under which cells are maintained during experiments. To overcome these problems, and also to increase the range of possible experimental conditions, we use a standard CO_2_/N_2_ 170 L tissue culture incubator that has been adapted by the addition of a water-chilled electron-multiplying charge-coupled device (EMCCD) camera with anti-mist optics and digital control of temperature and gas levels. This has been dubbed an Automated Longitudinal Luciferase Imaging Gas and Temperature-Optimized Recorder, or ALLIGATOR. The ALLIGATOR allows for substantially increased flexibility of bioluminescent imaging, both for high-throughput imaging of standard tissue culture plates (up to 6 x 96- or 384-well plates simultaneously) and also for non-standard tissue culture systems, such as perfused cells grown in microfluidic devices. This instrument also allows for imaging to occur under humidified conditions and with variable control of both CO_2 _and O_2_ partial pressure as well as temperature.

The protocol below describes a method for the bioluminescent recording of mammalian cell and tissue culture systems using an ALLIGATOR (henceforth referred to as 'bioluminescence incubator'). It should be noted, however, that the system would be well suited to bioluminescent imaging and also, with some modification, fluorescent imaging, in a number of other biological systems and contexts.

## Protocol

### 1. Seeding and Temperature Entrainment of Cells

NOTE: This protocol has been rigorously tested using primary and immortalized mouse fibroblasts expressing the PERIOD2::LUCIFERASE (PER2::LUC) fusion protein^4^. Adjustments may need to be made for experiments using other cell lines.

1. Prior to beginning cell culture, place the following into a 37 °C water bath to warm: phosphate-buffered saline (PBS) (pH 7.4), PBS supplemented with 0.68 mM ethylenediaminetetraacetic acid, pH 7.2 (PBS+EDTA), appropriate cell culture media, *e.g.*, Dulbecco's Modified Eagle's Medium (DMEM) supplemented with 10% fetal bovine serum (FBS), 100 U/mL penicillin and 100 µg/mL streptomycin, and 0.25% trypsin solution.
2. Dilute trypsin 1:3 with pre-warmed PBS+EDTA solution and return to the water bath.
3. Take a 125 cm^2^ flask of luciferase-expressing cells that are 70-90% confluent. Aspirate the media. Add 10 mL pre-warmed PBS.
4. Aspirate the PBS. Add 2.5 mL pre-warmed trypsin-EDTA and return the flask to a 37 °C incubator for 5 min.
5. Remove the cells from the incubator and view under a microscope. Once the majority of cells have detached from the bottom of the flask, continue to the next step. If most cells remain attached to the bottom of the flask, return it to the incubator until most cells are in suspension.
6. Add a further 7.5 mL of standard cell culture medium to the flask. Optionally, remove 1 mL of this for further passage of the cells, as required.
7. Count the density of the remaining cells using a hemocytometer and trypan blue. Dilute the cells further, as appropriate for that cell line. NOTE: For the experiments below, PERIOD2::LUC fibroblasts were seeded between 6 x 10^3^ cells/cm^2^ and 1 x 10^4^ cells/cm^2^ in either 96-well plates, 35 mm dishes, or channel slides.
8. Seed cells onto the tissue culture dishes or plates that will be used for recording. NOTE: Use of black sided, clear-bottomed plates is recommended as this allows culture health to be assessed before recordings begin whilst reducing interference between wells during recording. Where bioluminescence levels are extremely low, white dishes/plates should be used to maximize light detection, although please note that phosphorescence can lead to increased background signal that persists for several hours.
9. Return the plates to the incubator and allow monolayers to reach 100% confluence (approximately 7 days). NOTE: Once confluent, cell lines that inhibit contact can be maintained for up to three weeks prior to experimentation provided that the media is refreshed regularly (every 5-7 days).
10. Once confluent, synchronize cellular rhythms using applied temperature cycles (12 h at 32 °C, 12 h at 37 °C) for a minimum of 72 h immediately prior to experimentation^5^,^6^ (using a pre-programmed cycling incubator, controlled by a computer connected to the incubator through a serial RS-232 port). NOTE: The number of temperature cycles required for synchronization of cellular rhythms may vary between cells lines and may therefore need to be optimized for other cell lines. Acute pharmacological stimulation using forskolin or dexamethasone has also been used previously to synchronize cellular rhythms^7^,^8^.

### 2. NS21 Preparation

NOTE: NS21 is a serum-free supplement for the maintenance of neuronal and other cell cultures. It is a refinement of a similar supplement known as B27, which is commercially available and can be used as a serum replacement during circadian bioluminescence recordings^9^. Either supplement can be used interchangeably in the recording media for the experimental protocols described below. It is quite feasible and cost effective to make NS21 in-house, as in Chen *et al.*^10^, and as described here.

1. Equilibrate all components described in **Table 1** at room temperature for 1 h before beginning.
2. Dissolve 50 g bovine albumin in 324 mL basal medium (*e.g.*, neurobasal) on ice. Stir the mixture as little as possible.
3. Add all other components, stirring minimally after each component but still ensuring thorough mixing. Aim to dissolve all components within 90 min of starting.
4. Aliquot the final mixture and store at -20 °C until required. Avoid repeated freeze-thaw cycles. NOTE: The mixture is too viscous to filter at this stage, but will be sterile filtered when diluted with media before adding it to cells.

### 3. Recording Media Preparation

NOTE: A primary advantage of the bioluminescence incubator over other equipment for recording longitudinal bioluminescence is that, by virtue of being able to humidify the incubator and vary the partial pressures of O_2_ and CO_2_, it is possible to use a wider range of media conditions for recording bioluminescence — including conditions which more closely approximate the physiological niche occupied by different cell types *in vivo*. Below we describe the formulation of recording media adapted from Hastings *et al.*^9^, that we have used routinely with cultured fibroblasts and other cell types. The first is for sealed culture conditions (without gas exchange), and the second is for physiologically relevant conditions and should be used under humidified conditions with 5% CO_2_. Many other variations are both possible and advisable, depending on the exact application and cell type.

1. **MOPS-buffered high glucose media preparation** NOTE: Stock solution (1 L):DMEM powder (8.3 g/L), 0.35 mg/mL sodium bicarbonate*, *5 mg/mL glucose*, *0.02 M MOPS, 100 U/mL penicillin, 100 µg/mL streptomycin.
  1. Dissolve 8.3 g DMEM powder in 900 mL ultrapure water in a 1,000 mL measuring cylinder and stir for 30 min until all particles are dissolved.
  2. Add 50 mL of glucose solution (100 g/L), 20 mL of MOPS (1 M, pH 7.6), 10 mL of 100x pen/strep solution, and stir for a further 10 min.
  3. Add 4.7 mL 7.5% sodium bicarbonate solution and stir for a further 5 min.
  4. Adjust the media pH to 7.6 (if at room temperature) or 7.4 (37 °C) with HCl/NaOH.
  5. Add ultrapure water to produce a final volume of 1,000 mL.
  6. Sterilize by filtration through a 0.22 µm filter and store at 4 °C until required.
  7. Prior to experimentation, supplement an appropriate volume of stock solution with 10% serum, 2mM L-alanyl-L-glutamine, 2% NS21, and 1 mM luciferin to make a working stock. Pass this stock through a 0.22 µm sterile filter.
  8. Measure osmolality of the media using an osmometer.
    1. Turn on the osmometer.
    2. Calibrate the osmometer, first with ultrapure water. Add 50 µL water to the bottom of a measuring vessel. Ensure there are no bubbles in the liquid. Clip the vessel over the probe. Press 'Zero' and carefully lower the osmometer arm to the lowest point. Wait until reading is complete and green 'result' light is lit before raising the arm and removing the vessel.
    3. Repeat to calibrate the osmometer to 300 mOsmol/kg using 50 µL calibration standard. Press 'Cal' before lowering the arm.
    4. Measure the sample osmolality by adding 50 µL of media to the bottom of a measuring vessel and measure as above. Press 'sample' before lowering the osmometer arm.
  9. Adjust the osmolality to 350 mOsmol using 5 M NaCl.
2. **HCO_3_-buffered low glucose media (perfusion medium) preparation** NOTE: Stock solution (1 L): DMEM powder (8.3 g/L), 3.7 mg/mL sodium bicarbonate, 1 mg/mL glucose, 100 U/mL penicillin, 100 µg/mL streptomycin.
  1. Dissolve 8.3 g DMEM powder in 900 mL ultrapure water in a 1,000 mL measuring cylinder and stir for 30 min until all particles are dissolved.
  2. Add 50 mL of glucose solution (100 g/L), 10 mL of 100x pen/strep solution and stir for a further 10 min.
  3. Add 49.4 mL 7.5% sodium bicarbonate solution and stir for a further 5 min.
  4. Adjust the media pH to 7.6 (at room temperature) or 7.4 (37 °C) with HCl/NaOH.
  5. Add ultrapure water to give a final volume of 1,000 mL.
  6. Pass the solution through a 0.22 µm sterile filter and store at 4 °C until use.
  7. Prior to experimentation, supplement an appropriate volume of stock solution with 2% serum, 2 mM L-alanyl-L-glutamine, 2% NS21 and 1 mM luciferin to make a working stock. Pass this stock through a 0.22 µm sterile filter.
  8. Measure and adjust the osmolality to 350 mOsmol using 5 M NaCl, as for the MOPS-buffered media.

NOTE: Luciferin concentration should be determined empirically for each cell type and context. For further information see Feeney *et al.*^11^ Serum and NS21 (or B27 if used) concentrations can be varied depending on application. However, we do advise that, unless empirically tested to show otherwise, serum and NS21 (or alternative serum-free supplement) be used, as these promote cell survival and attachment. In cell lines that do not contact inhibit well, it may be necessary to lower the serum concentration in the recording media to prevent confounding effects of proliferation, which is also promoted by serum.

### 4. Recording

1. Prior to beginning the recording, place the appropriate working media stock (from step 3.1 or 3.2 depending on the experiment) in a 37 °C water bath to warm.
2. **Whilst the media is warming, focus the camera in the bioluminescence incubator.**
  1. Unscrew the water-impermeable, heated neutral density filter and set aside.
  2. Set the software to record a video with a high acquisition rate. Click "Acquisition | Acquisition Setup". Set 'Exposure time' to 0.2 s and kinetic cycle time to 0.3 seconds. Make sure that EM gain is turned off.
  3. Close the 'Acquisition Setup' window and click the "take video" icon.
  4. Using the focusing cylinder, rotate the camera lens until items on the shelf at the height to be recorded are clearly in focus in the video.
  5. Stop the video by clicking the 'stop video' icon.
  6. Screw the heated neutral density filter back into position; failure to do so will result in damage to the EM-CCD camera and/or fogging of the objective due to condensation.
3. Set O_2_, CO_2_, and temperature to the desired experimental conditions using the control panel on the front of the bioluminescence incubator. NOTE: If performing perfused cell culture proceed directly to Section 5 at this stage.
4. Remove the cells from the tissue culture incubator and aspirate off the media. Replace the media with pre-warmed working stock.
5. If the bioluminescence incubator is not to be humidified during the experiment, then seal the dishes and plates with a gas-impermeable seal. If the bioluminescence incubator is to be humidified, then it is not strictly necessary to seal dishes, although it is preferable to seal dishes with a small volume, such as 96-well plates, using a gas-permeable seal, as a small quantity of evaporation can otherwise still occur even in humidified incubators. NOTE: Humidification is achieved by filling the water tray at the base of the incubator.
6. Transfer the cells to the bioluminescence incubator, close the incubator door and secure incubator cover in place to form a light-tight seal.
7. **Select the recording parameters appropriate for the application.**
  1. Click "Acquisition| Acquisition Setup".
  2. In the 'Camera Setup' panel, set: 'Acquisition mode' to 'Kinetic'; 'Exposure time' to desired exposure time, in seconds (see note below); 'Accumulations' to 1; 'Kinetic Series length' to number of acquisition cycles desired; 'Kinetic cycle time' to desired imaging interval (ensure that this is greater than the exposure time); 'Shift speed' to 4.33 µsecs; 'Gain' to 1; and 'EM gain' to the desired level (see note below)
  3. In the 'Autosave' panel, set the file-type to .sif or .tif. Provide a file name and appropriate saving location.
  4. In the 'Spooling' panel, set the file-type to tiff. Provide a file name and appropriate saving location. Close the acquisition setup window.
8. Start recording by clicking the 'take signal' button. NOTE: As the bioluminescence incubator can be used for a large number of different cell and tissue types, all of which will have different levels of bioluminescence, the exposure time required to collect an appropriate bioluminescent signal will vary between experiments. It is also possible to vary the electron-multiplying (EM) gain of imaging in order to increase the magnitude of the signal collected. For new applications of unknown brightness, we suggest first taking a single image with a relatively short exposure time (around 10 min) without EM gain and subsequently adjusting both exposure time and EM gain until the desired recording conditions have been reached. In most cases, a mean signal of 10-20% of the maximum possible pixel intensity for the camera is a good level to aim for. Once an appropriate set of recording parameters has been reached, start longitudinal acquisition of images, as described above.

### 5. Perfused Tissue Culture (Optional)

NOTE: As described in the introduction, the bioluminescence incubator is suitable for imaging of non-standard tissue culture systems. This is exemplified in the development of a system for perfused cell culture.

1. Seed the cells onto single channel slides with Luer connectors with a channel depth of 0.6 or 0.8 mm, in DMEM with 10% FBS and pen/strep, as described in Section 1. Make the perfusion media as described in step 3.2.
2. Prior to recording, prepare tubing for the required perfusion system using 1 mm internal diameter (I.D.) ETFE tubing and 1 mm I.D. silicone tubing, elbow, male and female Luer fittings, as shown in Figure 2A. NOTE: The majority of the length of the tubing is ETFE tubing, with 2 cm sections of silicone tubing used to connect the ETFE tubing to the Luer fittings, which subsequently link into the channel slides.
3. Sterilize the tubing by flushing with 70% ethanol, followed by sterile PBS.
4. Remove the cell cultures in channel slides from the incubator. Aspirate the media and replace with pre-warmed perfusion media.
5. Fill a 20 mL syringe with the pre-warmed perfusion media.
6. Connect the tubing to the buffer slide (see Figure 2) but not to the cell-containing slide, and flush the tubing and slide with perfusion media (3-5 mL).
7. Connect the cell-containing slide and flush the whole system with a further 1 mL of media to remove any air bubbles.
8. Transfer the entire system to the bioluminescence incubator.
9. Fit the media-filled syringes to the syringe pump without disconnecting from the tubing and flush a further 1 mL of media through the entire system using the pump to ensure there are no air bubbles in the system.
10. Set the diameter on the pump to that of the syringe being used. NOTE: For the 20 mL syringes used here, the diameter is 19.13 mm.
11. Set the flow rate of the pump to 50 µL/h and start it running.
12. Ensure that there are no bubbles in any slides or tubing before the start of recording, as these will influence luciferase expression when they pass across the cell monolayer. If necessary, flush further media across the cells to achieve this.
13. Close the bioluminescence incubator door and continue as in step 4.7 to begin recording.

### 6. Treatment During Recording

NOTE: Sometimes it is desirable to treat cells midway through a recording, be it with pharmacological or hormonal agents. In such cases, it is imperative that the cells are handled with care to prevent the cellular oscillation from resetting during treatment. For this reason, it is of particular importance that the cells are maintained at a constant temperature, as this is a major entraining cue for cellular circadian rhythms^5^,^6^.

1. Prepare all components of treatment prior to stopping recording.
2. Prepare a chemical isothermal pad at 37 °C.
3. Stop the device recording, remove the dishes to be treated and place on the isothermal heat pad.
4. Treat the cultures as required and return to the bioluminescence incubator in the same location as before.
5. Re-start recording.

### 7. Analysis

NOTE: The bioluminescence incubator produces data in the form of a series of individual images. We primarily use Fiji^12^ to manage these images and then export the mean pixel intensity data for each region-of-interest (ROI) for further analysis.

1. Open the image stack in Fiji and adjust the brightness and contrast by clicking "Image| Adjust| Brightness/Contrast" and adjusting the sliding bars until images are within an appropriate range for viewing the  bioluminescent signal.
2. Select areas of interest using the ROI manager available under "Analyze| Tools| ROI Manager".
3. Export mean signal for the selected area by clicking "ROI manager| More| Multimeasure".
4. Copy the resulting data into analysis software.
5. Manually adjust the time base of data to the time interval of imaging, (*i.e.*, 15, 30 or 60 min intervals, described as 0.25, 0.5, or 1 h) rather than the image number provided by Fiji. NOTE: Further analysis can now be performed, such as determination of period. For this, the following equation is used to perform non-linear regression analysis: 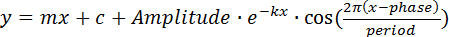 where *y* is the signal, *x* the corresponding time, *m* is the gradient of the trend line, *c* is the y intercept of the trend line, Amplitude is the height of the peak of the waveform above the trend line, *k* is the decay constant or rate of damping (such that 1/*k* is the half-life), phase is the shift in x of the cosine wave, and the period is the time taken for a complete cycle to occur^13^. R^2^ value is used as an indicator of goodness-of-fit.  Other analysis methods are also possible. The first 24 h of the acquisition should be excluded from analysis, as cellular bioluminesence may exhibit transient, non-circadian changes during this time. This is the result of an acute response to media change.

## Representative Results

This article outlines a protocol for the bioluminescent imaging of mammalian cells using an ALLIGATOR (bioluminescence incubator). This technique allows for flexibility of physical setup and extracellular conditions when imaging bioluminescent systems. Methods for both simple static tissue culture (Figure 1,** Supplementary Video 1**) and perfused cell culture (Figure 2, **Supplementary Video 2**) are described, but many other setups could be imaged using this system. All data were quantified using the methods described in Section 7.

Figure 1A shows an example video of a bioluminescence recording from 6 x 96-well plates containing PER2::LUC fibroblasts^4^. The outermost wells do not contain cells as these were not required for this experiment. Cells have undergone differential temperature entrainment, whereby they undergo either temperature cycle of 12 h, at 32 °C followed by 12 h 37 °C for 72 h or the converse (12 h, at 37 °C followed by 12 h, at 32 °C for 72 h), before being held at constant 37 °C for recording. 60 min exposures were taken every hour. Two of these conditions are quantified in Figure 1B.

Figure 2A shows a schematic of the setup of a system for perfused tissue culture. This consists of two channel slides connected with tubing. Media is driven across the cells by a syringe pump. The first of these slides acts as a gas permeable bubble trap (buffer slide) and contains no cells, with the second containing the cells from which bioluminescence is recorded. A representative recording video from this system is shown in Figure 2B. 15 min exposures were taken every 15 min. Here, cells are maintained either under standard perfusion conditions or in the presence of casein kinase inhibitor PF670462, which has been previously shown to lengthen circadian period and reduce the amplitude of clock gene expression rhythms in cultured mammalian cells^14^. The effect on PER2::LUC expression is shown in Figure 2C (top panel) against cells treated with the same concentration of drug under standard static cell culture conditions shown in Figure 2C (bottom panel), with quantification of period shown in Figure 2D. It is clear from this that treatment with PF670462 influences PER2::LUC expression under both sets of conditions. However, whilst cells treated with PF670462 under perfused conditions show period lengthening of approximately 3 h (3 ±0.9 h), cells in static conditions treated with the drug show substantially greater period lengthening to a period of >48 h. This can be fit by a damped cosine, as described in Section 7, (extra-sum-of-squares F test *versus* a straight line, p <0.0001). Interestingly, the magnitude of period lengthening under perfusion is much closer to that observed *in vivo*^14^.

**Figure:**
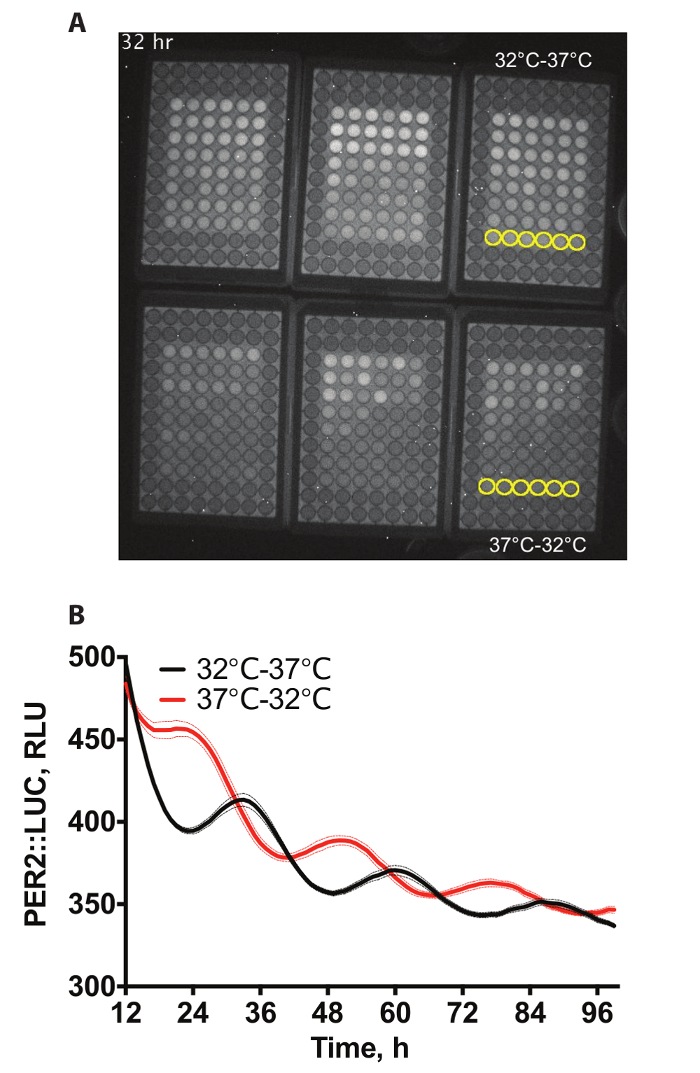

**Figure 1: Data example.** (**A**) Example video snapshot of bioluminescence from immortalized PER2::LUC in 6 x 96 well plates in the bioluminescence incubator. Wells to be quantified have been highlighted in yellow in the **Supplementary Video 1**. (**B**) Quantification of bioluminescence from A. Two sets of 3 plates of cells were entrained using temperature cycles (12 h, at 32 °C; 12 h, at 37°C) that were anti-phasic to each other to produce opposite phases of PER2 protein expression (n = 6, mean ± SEM). Please click here to view a larger version of this figure.

**Figure:**
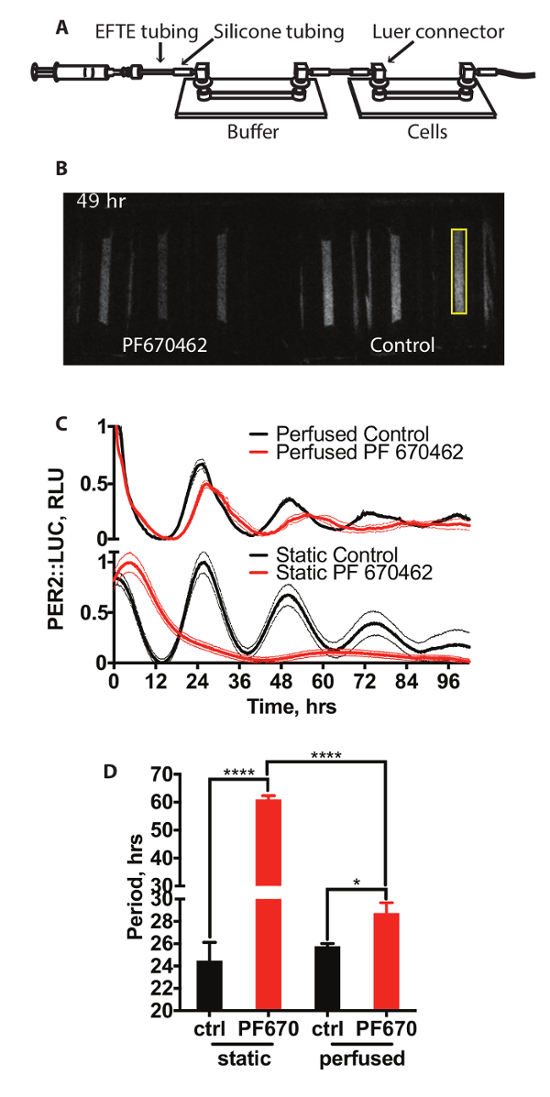

**Figure 2: Perfusion with CK1δ inhibitors. **(**A**) Schematic of perfusion system. (**B**) Example video snapshot of a bioluminescence recording from PER2::LUC cells under perfusion. A sample area of quantification is highlighted in yellow. (**C**) Quantification of bioluminescence of PER2::LUC fibroblasts under perfusion and in static conditions with and without 3 µM CK1 inhibitor PF670462 (n = 3, mean ± SEM). Bioluminescence has been normalized to minimum and maximum values. (**D**) Analysis of period (Two-way ANOVA, Holm-Sidak's multiple comparisons test). Please click here to view a larger version of this figure.

**Table:** 

**Component**	**Final Medium Concentration (μM)**	**Stock (mg/mL)**	**For 400 mL NS21**
Bovine Albumin	37	-	50 g
Catalase	0.01	-	50 mg
Glutathione	3.2	-	20 mg
Insulin	0.6	10	8 mL
Superoxide dismutase	0.077	-	50 mg
Holo-transferrin	0.062	-	100 mg
T3 (triiodo-L-thyronine)	0.0026	2	20 µL
L-Carnitine	12	-	40 mg
Ethanolamine	16	Liquid (1 g/ml)	20 µL
D(+)-Galactose	83	-	300 mg
Putrescine	183	-	322 mg
Sodium Selenite	0.083	1	280 µL
Corticosterone	0.058	2	0.2 mL
Linoleic Acid	3.5	100	0.2 mL
Linolenic Acid	3.5	100	0.2 mL
Lipoic Acid	0.2	4.7	0.2 mL
Progesterone	0.02	3.2	0.04 mL
Retinol Acetate	0.2	20	0.1 mL
Retinol, all trans	0.3	10	0.2 mL
D,L-alpha-Tocopherol	2.3	100	0. 2 mL
D,L-alpha-Tocopherol acetate	2.1	100	0.2 mL

**Table 1: NS21 Preparation.**

**Supplementary Video 1: Example video of bioluminescence from well plates**. Example video snapshot of bioluminescence from immortalized PER2::LUC in 6 x 96 well plates in the bioluminescence incubator. Wells to be quantified are highlighted in yellow. Please click here to download this file.

**Supplementary Video 2: Example video snapshot of a bioluminescence recording from PER2::LUC cells under perfusion. **A sample area of quantification is highlighted in yellow. Please click here to download this file.

## Discussion

The protocol described here is for mammalian cell culture, both under perfused and static conditions. However, the ALLIGATOR can easily be adapted to other model systems. Indeed, it has already been shown to provide an excellent platform for simultaneous monitoring of locomotion, sleep, and peripheral gene expression rhythms in *Drosophila melanogaster* maintained under constant darkness^15^*.* It is also noted that, depending on the application, camera types other than that mentioned here may be appropriate. We envisage that with the appropriate filters, a modified version of the current setup could in principal be used for fluorescence quantification.

The only applications for which the ALLIGATOR might not be appropriate are those for which particularly high spatial resolution is required, such as imaging of spatiotemporal organization of PER2::LUC expression in organotypic slices of the mammalian suprachiasmatic nucleus, or other small tissue slices.

The ALLIGATOR enables many experiments to be performed that hitherto have not been readily achievable by conventional recording techniques. Compared with current methods for the measurement of bioluminescence, the ALLIGATOR provides increased flexibility in both the type of cell culture dish or slide that can be used, the external media conditions, sensitivity, and processivity.

This is particularly relevant at a time when there is a move away from standard 2D cell culture models towards 3D organoid and flow culture systems. As such, it is anticipated that the ALLIGATOR will provide an adaptable method by which bioluminescence can be measured over many days and weeks under a broad range of conditions.

## Disclosures

No conflicts of interest exist.
